# Evaluation of the Co–Go–Me angle as a predictor in Class II patients treated with Herbst appliance and skeletal anchorage: a retrospective cohort study

**DOI:** 10.3389/froh.2024.1389628

**Published:** 2024-04-30

**Authors:** Antonio Manni, Marco Migliorati, Andrea Boggio, Sara Drago, Elena Paggi, Chiara Calzolari, Giorgio Gastaldi, Mauro Cozzani

**Affiliations:** ^1^ Department of Dentistry, Vita-Salute San Raffaele University, Milan, Italy; ^2^ Istituto Giuseppe Cozzani, La Spezia, Italy; ^3^ Department of Integrated Diagnostic and Surgical Sciences, University of Genoa, Genoa, Italy

**Keywords:** Class II, Herbst appliance, skeletal anchorage, gonial angle, TADs

## Abstract

**Introduction:**

A condylion–gonion–menton (Co–Go–Me) angle threshold of 125.5° has been introduced as a predictive parameter of cephalometric mandibular response in the orthopedic treatment of growing Class II patients with functional appliances, despite some contradictions in the literature. Considering the lack of studies evaluating the role of skeletal anchorage, this study aims to reassess the threshold of 125.5° in the Co–Go–Me angle as a useful predictor in growing skeletal Class II patients treated with acrylic splint Herbst appliance and two mini-screws in the lower arch (STM2).

**Methods:**

Thirty-five consecutively treated patients (20 males, 15 females; mean age, 11.37 years) with mandibular retrusion were classified into two groups according to their Co–Go–Me baseline values (Group 1, <125.5°; Group 2, >125.5°). The STM2 protocol involved the use of the MTH Herbst appliance with an acrylic splint in the lower arch and two interradicular mini-screws as anchorage reinforcement. Cephalometric analysis was performed by the same operator for each patient at baseline (T0) and at the end of the Herbst phase (T1). The effects of time and group on the variables were assessed by a repeated-measures analysis of variance. The primary research outcome was the difference between the groups in terms of mandibular responsiveness to treatment referred to as the relative difference (T1−T0) in Co_Gn.

**Results:**

The mean duration of the treatment was 9.5 months. No statistically significant differences between groups were detected at baseline, except from the expected SN/GoMe° (*p* < 0.001) and Co–Go mm (*p* = 0.028). No statistically significant changes between groups, which were caused by the treatment, were found considering the mandibular sagittal and vertical skeletal parameters. Similarly, no statistically significant differences were found in the dental changes between the high-angle and low-angle patients, apart from the upper molar sagittal position (*p* = 0.013).

**Discussion and conclusions:**

The 125.5° threshold in the Co–Go–Me value was not a reliable predictive parameter for the mandibular response in growing patients treated with the MTH Herbst appliance and lower skeletal anchorage. Due to its effective control in the sagittal and vertical planes, the STM2 technique might be an appropriate protocol to use in treating skeletal Class II patients, regardless of the growth pattern.

## Introduction

Class II is one of the most frequent malocclusions affecting approximately one-third of the world's population (1). The origin of this condition is either dental, skeletal, or both, but mandibular retrusion is the most common cause, even though it can also be produced by maxillary protrusion or a combination of them (2, 3). In addition to dental features, patients generally present a convex profile, which negatively affects the aesthetic appearance of the face (4).

Apart from a proper diagnosis, the success of a Class II orthopedic treatment depends on several variables, including the type of appliance, patient growth, and timing of intervention (5).

Among different types of functional appliances, the Herbst appliance is one of the most efficient due to the reduced need for compliance. This fixed appliance promotes mandibular advancement and reduces maxillary sagittal growth (6–8). In addition, it acts on the dentition through distalization of the upper arch and mesialization of the lower one. While skeletal effects are favorable, the typical dental compensations (i.e., lingual tipping of the upper incisors and buccal flaring of the lower ones and distalization of the upper molars and mesialization of the lower ones) caused by anchorage loss (8, 9) could reduce the overjet needed for a proper mandibular advancement, partially compromising the final treatment outcome (4).

These side effects could be overcome with the introduction of skeletal anchorage (10). The combination of Herbst appliance with a lower acrylic splint and two buccal temporary anchored devices (TADs) in the lower arch (STM2: Skeletal Therapy Manni Telescopic Herbst with two TADs) has already demonstrated to be effective in encouraging a significant mandibular advancement, limiting the undesired proclination of lower incisors (8).

In addition to the type of appliance, another crucial element that influences the outcome of this orthopedic treatment might be the timing of intervention. Generally, the time window around the growth peak is considered the best one for increasing the skeletal effects (11, 12).

Despite these predictive factors, great variability in individual response is known depending on the type of protocol and appliance (13–15), even with the same workflow (2, 5). Therefore, it is of great clinical interest to better define other predictors to expect a good or poor response to treatment. Patient growth patterns can influence orthodontic treatment (16); therefore, several cephalometric methods have been proposed to examine mandibular development. Among various methods (17–19), an important value is the condylion–gonion–menton (Co–Go–Me) angle determined by the ratio of the condylar axis to the base of the mandible (20). This element provides important information about the patient's verticality and growth pattern. According to Franchi and Baccetti (5), patients with a Co–Go–Me angle of less than 125.5° would respond positively to Class II functional jaw orthopedic therapy when applied at the peak of growth. Similarly, the study conducted by Baccetti et al. (21) showed a favorable increase in the mandibular length associated with a pre-treatment Co–Go–Me angle of less than 123°. This is correlated with the improved soft tissue and chin projection when the patient is treated with bonded Herbst, followed by fixed appliances. On the contrary, in the study conducted by Fleming et al. (22), no correlation was found between the Co–Go–Me angle and the skeletal and dentoalveolar responses to treatment with a Twin-Block appliance.

Given these apparent contradictions and the lack of studies considering the role of skeletal anchorage, this study aims to better evaluate whether or not the Co–Go–Me angle can also be considered an effective predictor in the orthopedic treatment of skeletal Class II patients treated with fixed functional appliance and mini-screws. In particular, the null hypothesis of this investigation is that the 125.5° threshold in the Co–Go–Me angle is not a predictive parameter for mandibular response in growing patients treated with MTH Herbst and two lower TADs (STM2).

## Materials and methods

### Study design and recruitment

This retrospective study was performed on a sample of 35 skeletal Class II patients (20 males, 15 females; mean age, 11.37 ± 1.82 years) with a convex profile, who were consecutively treated with the STM2 technique. The inclusion criteria for this investigation were as follows: bilateral angle Class II Division 1 malocclusion (≥1/2 cusp width), skeletal Class II (ANB ≥ 4°), overjet ≥4 mm, permanent or late mixed dentition, aesthetic improvement of the profile simulating a mandibular advancement, and signed informed consent. The exclusion criteria were as follows: the presence of a systemic disease, bone pathology, tooth agenesis or premature loss of permanent teeth, previous orthodontic treatments, and incomplete available records. All patients were evaluated and consecutively treated by the same operator with the main goal of maximizing the skeletal effects and limiting the protrusion of the lower incisors.

### Treatment protocol

A palatal expansion with a rapid palatal expander (RPE, A0620-13 Leone expansion screw, Sesto Florentino, Italy) was performed before the Herbst insertion in all patients because a transversal discrepancy was observed when simulating mandibular advancement. The activation protocol for the palatal expansion was one turn per day (0.2 mm for each activation) until the correction was completed.

**Figure 1:**
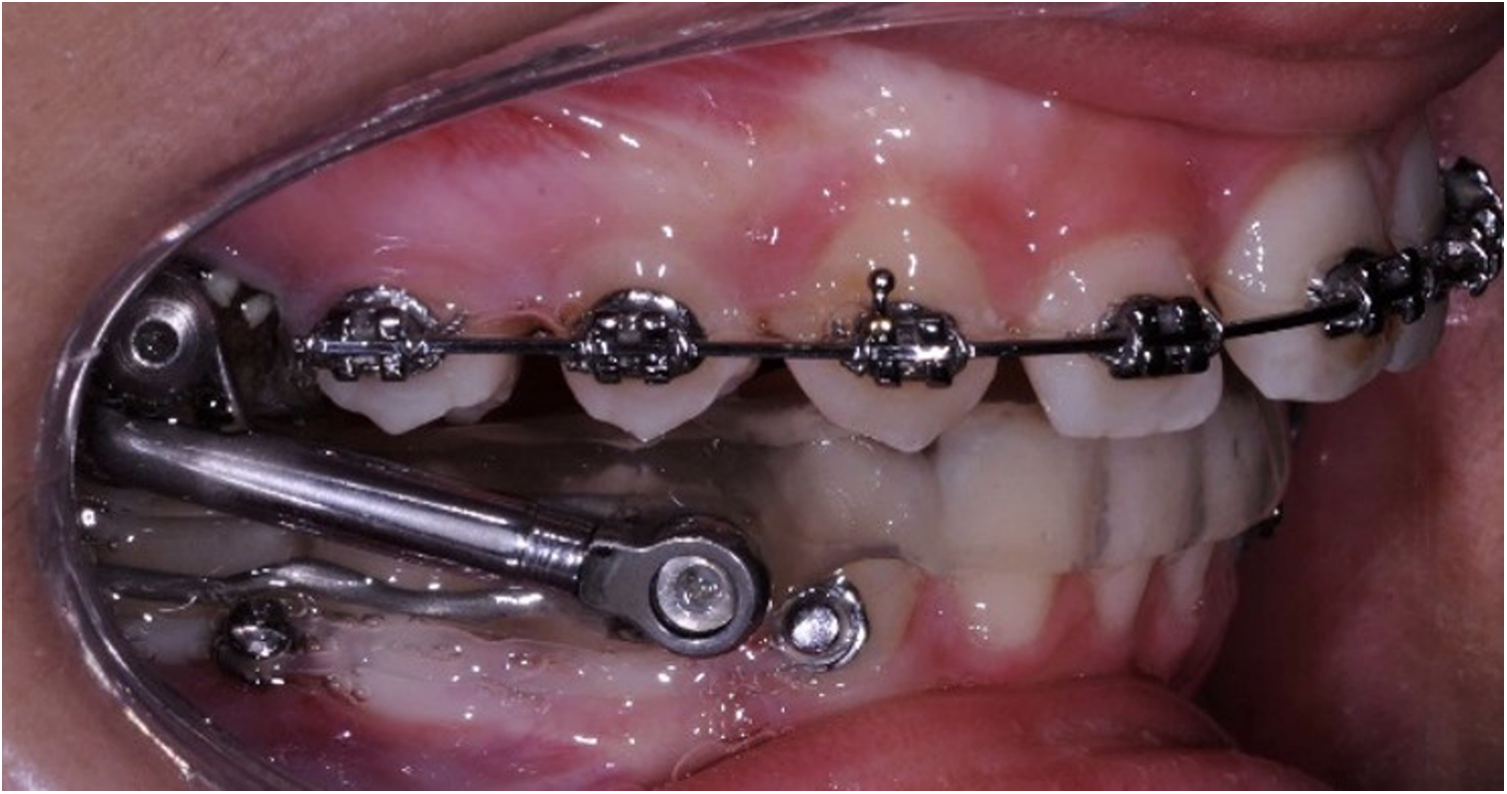
STM2 protocol: Herbst appliance with mini-screws in the lower arch used as anchorage reinforcement. The force applied to the screws is an elastic chain from the head of the mini-screws to a direct button applied on the canines.

The following STM2 protocol was then used: an MTH Herbst appliance with an acrylic splint (American Orthodontics, Sheboygan, WI, USA) was applied and initially activated with a mandibular advancement of 4 mm. At the same time, two TADs (length, 8.0 mm, diameter, 1.4 mm; Osstem Implant Co, Ltd., Seoul, South Korea) were placed in the lower arch only to act as anchorage reinforcement during the Herbst treatment (Figure 1). Based on bone availability, the insertion sites were between the first molar and the second premolar or between the two premolars (8) on the mucogingival line or in the attached gingiva. The TAD inclination was either perpendicular or tilted at 45° with respect to the alveolar bone depending on the clinical and anatomical conditions. The pre- and post-insertion radiographs of the interradicular implant sites were taken to check the distance of the TAD from the dental roots. All TADs were manually inserted without predrilling. The bilateral elastic chains (Memory Chain; American Orthodontics, Sheboygan, WI, USA) were stretched with a force of approximately 150 g from the head of the mini-screws to the direct buttons bonded on the buccal surface of lower canines as anchorage reinforcement. The elastic chains were changed at every appointment, supposing an average reduction of approximately 40% of the force after 1 month (23).

The mandible was advanced by gradual increments (2 mm every 2 months) until the full correction of Class II (molar and canine Class I relationship) was reached, but the appliance was never removed before 9 months.

### Cephalometric evaluation

Three dropouts were registered and excluded from the analysis. All the other patients completed the treatment. Lateral cephalograms were collected at baseline (T0) and at the time of Herbst appliance removal (T1). A modified cephalometric Pancherz analysis (24) was performed for each patient at T0 and T1. The considered parameters are shown in Table 1.

**Table 1:** Baseline characteristics of the whole population (*N* = 35).

	Co–Go–Me		*p-*value
	<125.5°	>125.5°	
*N*	19	16	
Age	11.53 ± 1.84	11.19 ± 1.83	0.250
Sex			
Male (%)	10 (52.6)	10 (62.5)	0.734
Female (%)	9 (47.4)	6 (37.5)	
Ms_Olp (mm)	49.26 ± 2.89	48.88 ± 3.63	0.638
Mi_Olp (mm)	48.21 ± 4.12	47.88 ± 3.40	0.776
A_Olp (mm)	71.55 ± 3.32	72.00 ± 3.60	0.413
Pg_Olp (mm)	71.87 ± 5.88	72.66 ± 4.25	0.570
Co_Olp (mm)	−5.84 ± 3.39	−6.75 ± 3.03	0.695
Co_Gn (mm)	99.55 ± 5.17	101.34 ± 5.04	0.410
Co_Go (mm)	50.92 ± 3.43	48.25 ± 3.43	0.047*
Is_Olp (mm)	78.26 ± 3.52	79.41 ± 3.37	0.263
Ii_Olp (mm)	70.95 ± 4.11	72.97 ± 4.26	0.136
Overjet (mm)	7.32 ± 2.45	6.44 ± 1.96	0.166
SNA (°)	81.00 ± 1.77	79.56 ± 2.99	0.372
SNB (°)	75.37 ± 1.85	74.50 ± 3.20	0.461
ANB (°)	5.00 (4.00, 9.50)	5.00 (4.00, 6.50)	0.829
U1/PP (°)	112.79 ± 9.15	115.81 ± 8.04	0.475
L1/MP (°)	102.47 ± 6.87	98.12 ± 8.22	0.101
Wits (mm)	2.50 (−7.00, 8.00)	2.00 (−0.50, 5.00)	0.869
SN/GoMe (°)	29.24 ± 4.56	36.19 ± 6.04	<0.001*

The results are expressed as mean **± **standard deviation or median (interquartile range) or frequency (percentage of subjects), *p*-value, Student's *t*-test *p*-value (age), Fisher's exact test *p*-value (sex), or Pr(F) of the repeated-measures ANOVA with group as a factor (cephalometric parameters).

The intraclass correlation coefficients (ICC) were calculated for the linear and angular measurements for 15 randomly selected cephalograms. Traces were performed by the same operator at one time after a 4-week interval. For angular measurements, the mean ICC value was greater than 0.98. For linear variables, the value was greater than 0.99.

The patients were classified into two groups according to their Co–Go–Me baseline value (<125.5° for Group 1 and >125.5° for Group 2). The observation periods for both group samples were all matched (39.00 weeks for Group 1, SD = 5.17, and 36.75 weeks for Group 2, SD = 5.00). No statistically significant difference was found.

The primary outcome of the research was the difference between the groups in terms of mandibular responsiveness to the treatment referred to as the relative difference (RD T1−T0) in Co_Gn.

### Sample size

The sample size estimation showed that 15 patients for each group achieved 80% power to detect the mean difference in Co–Gn of 2.00 mm, yielding a 2.50 estimated standard deviation of differences and a 0.05 significance level (alpha) using a *t*-test. The 2.0 mm threshold was chosen as a clinically significant difference (4). The standard deviation was estimated according to a previous test over the sample.

### Statistical analysis

The continuous variables are given as means ± standard deviations (SD) and medians with interquartile range (IR), whereas the categorical variables are given as number and/or percentage of subjects.

The baseline differences among ages were tested using the Student's *t*-test adjusted by using the Bonferroni method, while those between the sex compositions of the groups were tested by using the exact Fisher's test.

A repeated-measures analysis of variance (ANOVA) with group and time as factors was performed to assess the effects of time and group and any relative interaction in the cephalometric parameters. The differences with a *p*-value < 0.05 were selected as significant. The data acquired were analyzed in an R v3.4.4 software environment (25).

## Results

The mean duration of the treatment was 9.5 months (SD = 1.29). The baseline (T0) cephalometric values, mean age, and sex classification of both groups are shown in Table 1. No significant differences were found between the groups at the beginning of treatment, aside from SN/GoMe° (29.24 ± 4.56 Group 1 vs. 36.19 ± 6.04 Group 2; *p* < 0.001), which was significantly higher in patients with the Co–Go–Me angle of >125.5° and Co–Go (50.92 mm ± 3.43 mm Group 1 vs. 48.25 mm ± 3.43 mm Group 2; *p* = 0.047), which was statistically higher in patients with Co–Go–Me angle of <125.5°.

The results of the repeated-measures ANOVA with time and group as factors are shown in Table 2.

**Table 2:** Descriptive statistics and output of the repeated-measures ANOVA.

	*N*	Mean	95% lower CI	95% upper CI	*p*-value	Interaction
Ms_Olp (mm)						
Time					<0.001*	0.013*
T0	35	0	–	–		
T1	35	−2.43	−3.16	−1.70		
Group					0.638	
<125.5°	36	0	–	–		
>125.5°	34	0.60	−0.41	1.61		
Mi_Olp (mm)						
Time					<0.001*	0.579
T0	35	0	–	–		
T1	35	−3.47	2.78	4.17		
Group					0.776	
<125.5°	36	0	–	–		
>125.5°	34	−0.37	−1.43	−0.69		
Molar class (mm)						
Time					<0.001*	0.010*
T0	35	0	–	–		
T1	35	−12.67	−14.81	−10.54		
Group					0.685	
<125.5°	36	0	–	–		
>125.5°	34	1.00	0.01	1.99		
A_Olp (mm)						
Time					0.372	0.017*
T0	35	0	–	–		
T1	35	0.20	−0.31	0.71		
Group					0.413	
<125.5°	36	0	–	–		
>125.5°	34	1.01	0.13	1.88		

	*N*	Mean	95% lower CI	95% upper CI	*p*-Value	Interaction
Pg_Olp (mm)						
Time					<0.001*	0.504
T0	35	0	–	–		
T1	35	3.70	2.86	4.54		
Group					0.570	
<125.5°	36	0	–	–		
>125.5°	34	1.07	−0.50	2.64		
Co_Olp (mm)						
Time					0.063	0.649
T0	35	0	–	–		
T1	35	0.47	−0.01	0.95		
Group					0.695	
<125.5°	36	0	–	–		
>125.5°	34	−0.4	−1.16	0.36		
Is_Olp (mm)						
Time					0.994	0.809
T0	35	0	–	–		
T1	35	0.00	−0.91	0.91		
Group					0.263	
<125.5°	36	0	–	–		
>125.5°	34	1.44	0.44	2.44		
Ii_Olp (mm)						
Time					<0.001*	0.528
T0	35	0	–	–		
T1	35	3.29	2.61	3.96		
Group					0.136	
<125.5°	36	0	–	–		
>125.5°	34	2.09	1	3.18		

	*N*	Mean	95% lower CI	95% upper CI	*p*-Value	Interaction
Overjet (mm)						
Time					<0.001*	0.407
T0	35	0	–	–		
T1	35	−3.57	−4.36	−2.78		
Group					0.166	
<125.5°	36	0	–	–		
>125.5°	34	−0.65	−1.28	−0.02		
SNA (°)						
Time					0.026*	0.399
T0	35	0	–	–		
T1	35	−0.66	−1.19	−0.13		
Group					0.372	
<125.5°	36	0	–	–		
>125.5°	34	−0.77	−1.50	−0.04		
SNB (°)						
Time					<0.001*	0.634
T0	35	0	–	–		
T1	35	1.80	1.36	2,25		
Group					0.461	
<125.5°	36	0	–	–		
>125.5°	34	−0.66	−1.43	0.11		
ANB (°)						
Time					<0.001*	0.071
T0	35	0	–	–		
T1	35	−2.46	−2.81	−2.12		
Group					0.829	
<125.5°	36	0	–	–		
>125.5°	34	−0.11	−0.64	0.42		

	*N*	Mean	95% lower CI	95% upper CI	*p*-Value	Interaction
U1/PP (°)						
Time					0.790	0.245
T0	35	0	–	–		
T1	35	−0.34	−3.22	2.53		
Group					0.476	
<125.5°	36	0	–	–		
>125.5°	34	1.54	−0.33	3.41		
L1/MP (°)						
Time					0.352	0.352
T0	35	0	–	–		
T1	35	−0.50	−1.96	0.96		
Group					0.101	
<125.5°	36	0	–	–		
>125.5°	34	−4.33	−6.52	−2.14		
Wits (mm)						
Time					<0.001*	0.289
T0	35	0	–	–		
T1	35	−4.27	−4.87	−3.67		
Group					0.869	
<125.5°	36	0	–	–		
>125.5°	34	0.13	−0.75	1.01		
SNGoMe (°)						
Time					0.124	0.608
T0	35	0	–	–		
T1	35	−0.61	−1.38	0.15		
Group					<0.001*	
<125.5°	36	0	–	–		
>125.5°	34	6.53	5.07	7.99		

	*N*	Mean	95% lower CI	95% upper CI	*p*-Value	Interaction
CoGn (mm)						
Time					<0.001*	0.578
T0	35	0	–	–		
T1	35	4.04	3.21	4.88		
Group					0.410	
<125.5°	36	0	–	–		
>125.5°	34	1.48	0.08	2.88		
CoGo (mm)						
Time					<0.001*	0.663
T0	35	0	–	–		
T1	35	2.51	1.45	3.58		
Group					0.047*	
<125.5°	36	0	–	–		
>125.5°	34	−2.34	−3.17	−1.51		

*N*, number of observations; mean, mean difference; 95% lower CI, 95% lower confidence interval of the difference; 95% upper CI, 95% upper confidence interval of the difference; *p*-value, repeated-measures ANOVA Pr(F) value of the univariate effect for time and group, respectively; and interaction, repeated-measures ANOVA Pr(F) value of the group: time interaction.

Considering sagittal skeletal changes, no significant differences were observed between the groups in SNA° (−0.89 ± 1.27 Group 1 vs. −0.41 ± 1.98 Group 2; *p* = 0.399), SNB° (1.97 ± 1.37 Group 1 vs. 1.74 ± 1.55 Group 2; *p* = 0.634), ANB° (−2.86 ± 1.22 Group 1 vs. −2.15 ± 1.03 Group 2; *p* = 0.071), and Wits (−4.64 mm ± 1.82 mm Group 1 vs. −3.97 mm ± 1.85 mm Group 2; *p* = 0.289). The value representing the dimension of the mandibular body (Co_Gn) showed an increase in both groups (4.21 mm ± 2.72 mm in Group 1 and 4.03 mm ± 2.10 mm in Group 2; *p* = 0.578). Moreover, although not significant, the group with the Co–Go–Me angle of >125.5° showed a greater advancement of the pogonion (Pg_Olp) at the end of treatment in comparison with the group with the Co–Go–Me angle of <125.5° (3.34 mm ± 2.34 mm for Group 1 vs. 4.34 mm ± 2.41 mm for Group 2; *p* = 0.504). The maxillary bone base length (A-OLPmm) indeed slightly reduced in Group 1 (−0.39 mm ± 1.45 mm) and increased in Group 2 (0.82 mm ± 1.39 mm).

Considering the vertical skeletal changes, there was an increase in the mandibular ramus length (Co_Go) in both groups (2.71 mm ± 3.00 mm in Group 1 and 2.47 ± 3.45 in Group 2), but the difference was not statistically significant (*p* = 0.663). Similarly, there was a decrease in the mandibular plane angle (SN/GoMe°) in both groups (−0.39 ± 2.31 Group 1 vs. −0.81 ± 2.34 Group 2), but the difference was also not statistically significant (*p* = 0.608).

With regard to the dental parameters, there were no statistically significant changes between the groups in the upper incisor sagittal position (Is_Olp) (−0.11 mm ± 2.97 mm Group 1 vs. 0.12 mm ± 2.55 mm Group 2; *p* = 0.809) and inclination (U1/PP) (1.33 ± 9.36° Group 1 vs. −2.12 ± 7.77° Group 2; *p* = 0.245). Similarly, no statistically significant changes between the groups were registered in the lower incisor sagittal position (Ii-Olp) (3.50 mm ± 1.84 mm Group 1 vs. 3.06 mm ± 2.24 mm Group 2; *p* = 0.528) and inclination (L1/MP) (0.08 ± 4.43° Group 1 vs. −1.50 ± 4.46° Group 2; *p* = 0.352) and in the lower molar sagittal position (Mi_Olp) (3.67 mm ± 1.88 mm Group 1 vs. 3.26 mm ± 2.35 mm Group 2; *p* = 0.579). Conversely, the upper molar sagittal position (Ms_Olp) showed a statistically significant difference between the groups (−3.31 mm ± 2.38 mm Group 1 vs. −1.50 mm ± 1.62 mm Group 2; *p* = 0.013).

In this study, nine TADs failed and were immediately reinserted without significant consequences for the treatment. No significant issues were encountered during the treatment, except for some discomfort in the early stages of adaptation to the Herbst appliance. Three dropouts were registered: one related to medical issues independent of orthodontic treatment and two related to poor oral hygiene during treatment. These dropouts were excluded from the whole analysis.

## Discussion

The goal of orthopedic treatment in patients with skeletal Class II and mandibular retrusion is to increase the projection of the pogonion, thereby reducing the convexity and improving the aesthetics of the profile. When evaluating the mandibular response to functional treatment, it could be useful to assess the skeletal divergence. In fact, the vertical pattern may affect the condylar growth and chin projection (26). Generally, hyperdivergent patients exhibiting a clockwise rotation of the mandible might be more difficult to treat than hypodivergent patients who exhibit a counterclockwise rotation pattern. Therefore, Baccetti and Franchi (21, 5) considered the Co–Go–Me angle as a decisive parameter in predicting a good or poor mandibular response to orthopedic treatment. According to these authors, the presence of a reduced Co–Go–Me angle (i.e., Co–Go–Me angle of <125.5°) is associated with a greater mandibular length gain when using functional appliances. Although other studies confirmed the role of the Co–Go–Me angle as a useful predictor and reported the possible occurrence of a greater chin advancement with a smaller angle (27, 28), the threshold of 125.5° in the Co–Go–Me angle could not be confirmed in the present study as a valid response predictor when Class II patients were treated with acrylic splint Herbst appliance with two lower TADs as anchorage reinforcement. In fact, Groups 1 and 2 showed an increase in pogonion projection (Pg_Olp) (i.e., 3.34 mm ± 2.34 mm and 4.34 mm ± 2.41 mm, respectively. The difference was not statistically significant (*p* = 0.504). Similarly, Group 1 had an increased Co_Gn value of 4.21 mm ± 2.72 mm, while Group 2 had an increased Co_Gn value of 4.03 mm ± 2.10 mm. Thus, both groups showed an increase in the mandibular body length after treatment with the acrylic splint Herbst appliance and two lower TADs. The Co–Go–Me angle value was, however, not decisive as regards the statistically significant difference between the two groups (*p* = 0.578). In confirmation, both groups showed a reduction in the ANB angle (−2.86 ±** **1.22 in Group 1 and −2.15 ±** **1.03 in Group 2) and Wits (−4.64 mm ±** **1.82 mm in Group 1 and −3.97 mm ±** **1.85 mm In Group 2), but the difference was not statistically significant (*p* = 0.071 and *p* = 0.289, respectively). Finally, considering the mandibular ramus length (Co_Go), an increase was obtained in both groups (2.71 ± 3.00 for Group 1 and 2.47 ± 3.45 for Group 2), but the difference was also not statistically significant (*p* = 0.663).

Comparing these results with those of the meta-analysis conducted by Yang (29) on the effects of the Herbst appliance on the sagittal position of the mandibular base (Pg_OLp), a greater Pog advancement was observed in the present study [3.34 mm ± 2.34 mm (Group 1) and 4.34 mm ± 2.41 mm (Group 2)] vs. the average of 1.45 mm found by the author. This confirmed the conclusions of Al-Dboush (30), stating that the combination of the Herbst appliance with TADs might increase the effectiveness of the traditional orthopedic treatment, thereby leading to a better projection of the chin, regardless of the growth pattern. On one hand, this outcome could be explained by greater control in the sagittal position of the lower incisors with skeletal anchorage, preserving the overjet needed for mandibular advancement (31). On the other hand, good control of the vertical dimension was also observed probably due to the presence of the acrylic splint, which allowed a slight reduction in skeletal divergence not only in Group 1 (SN/GoMe°=−0.39 ± 2.31°) but also and especially in Group 2 (SN/GoMe°=−0.81 ± 2.34; the difference was not statistically significant, *p* = 0.608). Different from other Class II functional appliances, the presence of the splint, which limited the extrusion of the lower molars, probably prevented the clockwise rotation of the occlusal plane. The result led to an effective control of the skeletal divergence (32, 8) with a greater sagittal projection of the chin in hyperdivergent patients as well.

Considering the dental parameters, no statistically significant differences were observed among the groups in the maxillary incisor sagittal position (*p* = 0.809) and inclination (*p* = 0.245). A similar condition was observed for the lower incisor sagittal position (*p* = 0.528) and inclination (*p* = 0.352).

Moreover, no statistically significant differences were observed among groups in the mandibular sagittal position (*p* = 0.579), while the maxillary molars distalized more in Group 1 (Ms-Olp = −3.31 mm ± 2.38 mm) than in Group 2 (−1.50 mm ± 1.62 mm; *p* = 0.013).

This could be explained considering that forces related to the Herbst appliance normally tend to mesialize the lower arch and distalize the upper one. In the STM2 technique, however, due to the mandibular anchorage reinforcement, most of the forces are transferred to the upper molars. In low-angle patients, these applied forces might be generally higher because of the greater muscular component and more horizontal because of the orientation of the hinges relative to the occlusal plane. The result might be a slightly greater distalization of the upper arch, not only onto the anchorage unit (upper molars) but also at the level of the upper incisors (Is-OLP mm = −0.11 ± 2.97 Group 1; 0.12 ± 2.55 Group 2, even though the difference is not significant, *p* = 0.809) and of the A point (A-OLP mm = −0.39 ± 1.45 Group 1; 0.82 ± 1.39 Group 2; *p* = 0.017). Although this increased distalization may result in a statistically significant difference in the molar relationship between the two groups, the magnitude of this difference appears to be of little clinical significance.

The reason why the 125.5° threshold in the Co–Go–Me angle seems to be not decisive in assessing the mandibular response when the STM2 protocol is applied might be related to the applied therapy itself, which was different from the traditional one described in the article of Franchi and Baccetti (5). The STM2 technique involves the use of the telescopic Herbst associated with skeletal anchorage, elastic chains, and acrylic splint, with the main benefit of being represented by good control in both sagittal and vertical planes. A preliminary evaluation of the Co–Go–Me angle remains desirable in daily clinical practice in the treatment of skeletal Class II malocclusion. This parameter indeed makes it possible to predict the direction of the mandibular growth and preview the outcome of orthodontic therapy in cases where the STM2 technique is not adopted. Moreover, even when the orthodontist decides to use the Herbst appliance associated with skeletal anchorage, an evaluation of this parameter may be appropriate for the management of the occlusal plane during the second phase of treatment with multibracket therapy in order to achieve a proper occlusal result.

In the present study, nine TADs failed and were immediately reinserted without significant consequences for the treatment. This was in accordance with the mean failure rate reported in the current literature (33, 34). Generally, the acrylic splint Herbst appliance is associated with few emergencies, complications, and failures (35, 36). In this case, no significant issues were encountered during the treatment.

Despite the promising results, a larger sample size and more studies are necessary to confirm and better evaluate the success rate of such a protocol.

Moreover, a limitation of the study lies in the short-term evaluation of the treated patients, that is, only the Herbst phase was considered, even though the following one with multibracket appliances might affect the aesthetic outcome of the whole treatment. A long-term evaluation would also be necessary to assess the stability of the results. Finally, considering that the individual response could be influenced not only by the mean age at baseline but also by the skeletal maturation of the subjects, some maturity indicators could be considered in further evaluations.

## Conclusion

This study on Class II skeletal malocclusion in growing patients treated with Manni telescopic Herbst appliance combined with lower mini-screws and elastic chains (STM2 technique) led to the following conclusions:

• The 125.5° threshold in the Co–Go–Me value is not a predictive parameter for the mandibular response in growing patients treated with an MTH Herbst appliance and two lower TADs (STM2).
• No statistically significant differences can be found in the mandibular sagittal and vertical skeletal changes between the high-angle and low-angle patients treated with the STM2 technique.
• No statistically significant differences can be found in the dental changes between the high-angle and low-angle patients treated with the STM2 technique, aside from the upper molar sagittal position.
• Due to its effective control in the sagittal and vertical planes, the STM2 technique might be an appropriate protocol in the treatment of skeletal Class II patients, regardless of the growth pattern.

## Data Availability

The original contributions presented in the study are included in the article/Supplementary Material. Further inquiries can be directed to the corresponding author.
